# Assessment of the Psychometric Characteristics of the Italian Version of the Nurse Manager Actions Scale

**DOI:** 10.3390/nursrep13030102

**Published:** 2023-09-01

**Authors:** Marzia Lommi, Rosario Caruso, Gianluca Conte, Arianna Magon, Barbara Porcelli, Alessandro Stievano, Gennaro Rocco, Ippolito Notarnicola, Laura Sabatino, Roberto Latina, Maddalena De Maria, Emanuele Di Simone, Anna De Benedictis, Raffaella Gualandi, Daniela Tartaglini, Dhurata Ivziku

**Affiliations:** 1 Unit Care to the Person, Local Healthcare Authority Rome 2, 00159 Rome, Italy; marzia.lommi@aslroma2.it (M.L.); barbara.porcelli@aslroma2.it (B.P.); 2 Health Professions Research and Development Unit, IRCCS San Donato Hospital, San Donato Milanese, 20097 Milano, Italy or rosario.caruso@unimi.it (R.C.); gianluca.conte@grupposandonato.it (G.C.); arianna.magon@grupposandonato.it (A.M.); 3 Department of Biomedical Sciences for Health, University of Milan, 20133 Milano, Italy; 4 Department of Clinical and Experimental Medicine, University of Messina, 98100 Messina, Italy; alessandro.stievano@unime.it; 5 Department of Biomedical Sciences, Catholic University “Our Lady of Good Counsel”, 1000 Tirana, Albania; g.rocco@unizkm.al (G.R.); i.notarnicola@prof.unizkm.al (I.N.); or maddalena.demaria@uniroma2.eu (M.D.M.); 6 INAIL Istituto Nazionale per L’assicurazione Contro Gli Infortuni sul Lavoro, 00192 Rome, Italy; lau.sabatino@inail.it; 7 Department of Health Promotion, Mother and Child Care, Internal Medicine and Medical Specialities, University of Palermo, 90128 Palermo, Italy; roberto.latina@unipa.it; 8 Department of Biomedicine and Prevention, University of Rome Tor Vergata, 00133 Rome, Italy; 9 Department of Clinical and Molecular Medicine, Sapienza University, 00185 Rome, Italy; emanuele.disimone@uniroma1.it; 10 Clinical Directory, Fondazione Policlinico Universitario Campus Bio-Medico, 00128 Rome, Italy; a.debenedictis@policlinicocampus.it; 11 Department of Healthcare Professions, Fondazione Policlinico Universitario Campus Bio-Medico, 00128 Rome, Italy; d.tartaglini@policlinicocampus.it; 12 Società Italiana per la Direzione e il Management delle Professioni Infermieristiche (SIDMI), 00198 Rome, Italy

**Keywords:** NMA scale, autonomy, Mokken scale analysis, psychometric testing, nurses

## Abstract

Nurse managers play a vital role in healthcare organizations, wielding the ability to substantially enhance work environments, foster nurses’ autonomy, and bolster retention within workplaces. In this context, this study focuses on the Nurse Manager Actions scale, aiming to evaluate its items’ scalability as well as the scale’s validity and reliability among nurses and nurse managers operating within the Italian healthcare context. The study protocol was not registered. To ensure linguistic and cultural alignment, an iterative and collaborative translation process was undertaken. Subsequently, a multi-center cross-sectional design was adopted. Using a web-survey approach, data were collected among 683 nurses and 188 nurse managers between August 2022 and January 2023. The Nurse Manager Actions scale was found to be a valid and reliable instrument in Italian after a Mokken Scale Analysis. For nurses (*H^T^* = 0.630, Molenaar–Sijtsma rho = 0.890), the scale included 6 items, while 11 items were confirmed for nurse managers (*H^T^* = 0.620, Molenaar–Sijtsma rho = 0.830). Nurse Manager Actions scale scores were correlated with increased satisfaction and decreased intention to leave for both nurses and nurse managers. The employed validation process enhanced the scale validity for use in Italy and provided a model for other researchers to follow when assessing similar measures in different populations. Measuring and empowering nurse manager actions in work contexts is essential to improve the general well-being and retention of nurses, especially in the current nursing shortage.

## 1. Introduction

Nurses represent the largest workforce in healthcare organizations, and an important shortage is expected in the coming years [1]. To face this challenge, multiple efforts are required. On one side, it is necessary to increase the attractiveness of the nursing profession and promote uniform nursing education across nations [1,2]. On the other hand, it is essential to strengthen the current nursing workforce, invest in continued education, improve working conditions, create better work contexts, and enhance well-being to retain nurses [2].

Work environments have a significant impact on nurses’ well-being and retention. Disorganized work processes [3], reduced resources, and increased workloads [4] can lead to nurses experiencing stress, emotional exhaustion, reduced performance or engagement, turnover intentions, or absenteeism [5,6]. On the other hand, good working environments that enhance nursing professional autonomy and professionalism [7,8] and supportive management can help nurses to increase productivity [9], find meaning in their work [10], and maintain well-being [11]. Therefore, healthcare organizations should foster a supportive workplace culture that enables nurses to provide high-quality care while feeling valued and satisfied in their roles.

Nurse managers are in a key position to enhance supportive work environments for nurses. Through their leadership styles and actions, nurse managers can significantly affect nurses’ work and render work contexts favorable or not to improve nursing performance and autonomy. Nurse managers’ relational, participative, and transformational leadership styles stimulate personal development and encourage autonomy among nurses [7,12,13,14,15,16,17,18]. Additionally, nurse manager’s actions related to delegating authority and responsibility for decision-making [18], involving nurses in unit operation decisions [13,18], and supporting competence development [14,16] can empower nurses. This ultimately leads to improved coping, increased motivation and satisfaction, greater commitment to the organization, improved well-being, and improved retention [7,10,11,12,13,14,15,16,17,18]. Therefore, it is important for nurse managers to be good leaders, coaches, and counselors for their teams [19].

While nursing literature extensively discusses leadership styles, there is comparatively less exploration of managerial actions. Nurse manager actions encompass the activities and functions undertaken by a nurse manager to ensure the effective operation of the department and the delivery of high-quality patient care by influencing others toward achieving a common goal [14]. Advancing the understanding of nursing leadership and examining the dynamics of nurse manager actions and their potential interplay with other leadership characteristics [20] is key to improving nurses’ professional autonomy.

Nurses’ professional autonomy is a core concept in nursing [21]. In work settings, it impacts both nursing and patient care outcomes [10,13,14,17]. When nurses have autonomy at work, they feel more empowered, competent, and able to make independent decisions [7]. They also perceive greater well-being [11], job satisfaction [7,14,21,22,23], occupational commitment [9], and have a higher intention to stay and be recruited [11,14,22,23,24]. Patients benefit from the nurses’ autonomy as they experience greater safety, satisfaction, and better care quality [7,14,18,21,22,23]. Thus, promoting and supporting nurses’ autonomy in the workplace is essential to achieve optimal outcomes for all stakeholders involved.

In comparison to other European countries, the development of the nursing profession in Italy has faced hindrances arising from factors like limited recognition of professional status, inadequate institutional support, and the enduring dominance of the medical field. These factors have collectively led to curtailed autonomy for nurses [24,25]. Italian nurses commonly perceive their autonomy to be restricted, necessitating approval from physicians or managers even for critical decisions [26], and this pattern varies significantly across diverse care settings [27]. Given the recent transformations in the Italian healthcare system [28], it becomes increasingly crucial to delve comprehensively into the concept of autonomy within nursing practice. Additionally, it is important to explore how nurse managers could improve its enhancement within their nursing teams.

In the nursing literature, nurses’ autonomy has been extensively studied from the viewpoint of nurses [22]. Only a few studies have focused on nurse managers and their role in increasing nurses’ autonomy [13,14,22]. Recognizing the significance of nursing autonomy in healthcare settings and acknowledging the substantial impact of nurse managers’ actions, there is a clear advantage in expanding the scope of leadership research to encompass a more comprehensive examination of this subject. Conducting additional studies in this domain and delving deeper into the intricate role of nurse managers could yield valuable insights.

To bridge this research gap, the literature on nursing leadership was explicitly searched, and it identifies a valid and reliable measurement tool associated with nurse managers’ actions and the concept of nurses’ autonomy [29]. More precisely, we found only one appropriate tool for our study, the Nurse Manager Actions scale (NMAs) [13,30]. This scale was used in healthcare settings and nurse managers samples to evaluate nurse manager actions that increase nurses’ autonomy [13,14,30]. No studies have been conducted in Italy to investigate these dynamics within healthcare environments, and the instrument was unavailable in Italian. Therefore, before analyzing such relationships among nurses and nurse managers, a psychometric testing of the scale in Italian by also considering its items’ scalability is required to pave the way for descriptive or experimental studies.

Item scalability refers to the degree to which a particular item on a scale is related to the overall construct being measured by reflecting the underlying concept or idea that the scale is trying to measure [31]. An item with high scalability is considered to be a good indicator of the construct, while an item with low scalability may not be a good fit and should be removed from the scale. Scalability analysis is often used to develop and validate scales to ensure that the items are measuring the intended construct and are reliable and valid measures of that construct. This approach has to be considered preliminary to further investigations involving factor analyses and can be achieved by employing Mokken Scale Analysis (MSA) [32,33]. MSA provides a systematic method to assess item scalability, allowing researchers to objectively evaluate the alignment between items and the underlying construct.

Therefore, driven by the rationale that thorough evaluation of MSA, the NMAs has not ever been tested and the scale was not validated in Italian, this study aimed to evaluate its items’ scalability as well as the scale’s validity and reliability among nurses and nurse managers operating within the Italian healthcare context. To test further the construct validity of the scale, we used the hypothesis testing approach. In this regard, satisfaction and intention to leave are two important indicators related to nursing retention in work contexts and the profession. The extant body of literature has consistently documented positive relationships between nurses’ autonomy at work and job satisfaction and reduced turnover intentions [7,11,14,21,22,23]. Consequently, anticipated results involve discovering analogous relationships between the scores of NMAs and the levels of satisfaction and turnover intentions exhibited by both nurses and nurse managers by following these specific hypotheses: (a) Higher scores on the NMAs are correlated to greater satisfaction with the role, leadership, multidisciplinary teams, and organization among nurses and nurse managers; (b) higher scores on the NMAs are correlated to reduced intention to leave the unit, hospital, and profession among nurses and nurse managers.

## 2. Materials and Methods

### 2.1. Design

After having performed an iterative and collaborative translation process [34], we employed a multi-center cross-sectional design.

### 2.2. Collaborative and Iterative Translation

The translation and adaptation process of the NMAs from English to Italian was conducted following the methodological steps outlined by Douglas and Craig [34].

The research instrument used in this study went through a five-stage process: pre-translation, initial translation, pretesting, review, and administration. In the pre-translation stage (establish equivalence), a team of experts was involved in determining the conceptual definitions of the items in the instrument, focusing on three types of equivalences: category, functional, and construct. Category equivalence refers to similarities in the labels used to describe phenomena across cultures or languages, functional equivalence relates to the accuracy of the instrument in measuring the same construct across different cultural or linguistic backgrounds, and construct equivalence pertains to the similarity of underlying meaning or concepts being studied across cultures or languages. Eleven experts were involved in this phase, including a psychologist, four nurse managers, and six staff nurses. The experts had over 10 years of work tenure and were acknowledged in different healthcare settings.

After confirming the equivalence of the items, an independent, parallel translation into Italian was conducted by a translator with experience in translations of self-report questionnaires and a bilingual nurse researcher proficient in English and Italian. A review meeting involving the translators, experts, and authors was conducted to resolve any inconsistencies, ensure the accuracy of the translation of the items, and decide on the final version of the instrument in the Italian language.

The pretesting involved the authors who did not take part in translating the scale. They were asked to provide feedback about their understanding of the meaning of items, the ease of comprehension, clarity, and comprehensiveness. Any issues identified were discussed with the review team, and changes were incorporated into a decisive Italian version. The final version of the translated instrument in Italian is included in the Appendix A, with separate tables for the nurse (Table A1) and nurse manager versions (Table A2).

### 2.3. Sample and Setting of the Cross-Sectional Study

This cross-sectional study investigated nurse managers’ actions to promote nurses’ autonomy in various healthcare settings across different regions of Italy. The study included hospital units, outpatient services, theatre rooms, intensive and semi-intensive care units, as well as community settings such as community care centers, public healthcare services, home care, and community homes.

The principal investigators promoted the study through the network of the Italian Scientific Society for the Direction and Management of Nursing (SIDMI). Nurse executives who expressed interest in the research engaged local contact persons to facilitate the study’s introduction, commencing with nurse managers. Upon managerial assent to participation, the local contact individuals introduced the study to the nursing teams operating under the purview of the managers at each distinct study center, thereby proactively fostering their active involvement. A total of 871 participants working in 22 healthcare organizations from the northern, central, and southern regions of Italy participated in the study.

The eligibility criteria were the same for both nurses and nurse managers. Registered nurses and nurse managers were required (a) to be employed by a public or private healthcare organization, (b) work collaboratively with other nurses or nurse assistants in a team, (c) have at least one year of work tenure in the service, and (d) willingly consent to take part in the research. The exclusion criteria included (a) being a freelance registered nurse, (b) working independently without team collaboration, (c) having less than one year of experience in the service, (d) not being assigned to a stable work setting, (e) returning to the service less than six months after an extended absence, or (f) declining to participate in the study.

The sampling method employed was convenience-based, and participation was voluntary and anonymous. In the process of selecting units for study inclusion, a twofold strategy was employed to ensure participation, encompassing both the nurse manager and a mandated minimum of five nurses per unit. Local facilitators maintained a weekly communication channel with the principal investigators to monitor the progression of participation rates and proactively implement strategies to enhance engagement whenever deemed appropriate.

However, determining a minimum sample size was crucial to conducting a meaningful MSA that ensured the statistical reliability and validity of the study results. MSA is a non-parametric method used to evaluate the construct validity of a scale based on the principles of Item Response Theory. Unlike classical test theory methods, MSA assumes that the scale items form a hierarchical structure, where stronger items are endorsed by respondents who also endorse weaker items. The recommended minimum sample size for conducting MSA is 100 cases (respondents) with at least 10 observations (responses) per item [27,28]. This suggested minimum sample is determined from comprehensive simulation studies, which also incorporate sensitivity analyses [32,33]. Thus, with 11 items in the MSA, a minimum sample size of 110 participants per analysis is required. However, it is noteworthy that efforts were made in this study to surpass this minimum requirement and secure larger sample sizes to attain more accurate and robust results in the MSA analysis. For instance, Sijtsma and Molenaar conducted simulations to compare the performance of different item selection criteria in MSA under different sample sizes [33]. They found that the accuracy of the item selection criteria varied depending on the sample size, and larger sample sizes were recommended to ensure accurate results. Therefore, authors should aim for a larger sample size than the minimum requirement in studies using MSA when possible.

### 2.4. Data Collection

Data were collected between August 2022 and January 2023 through an online survey administered via the Google Forms platform. The survey explained the study’s goals and participation procedure and included a data handling section and informed consent. The local contact persons disseminated the survey link through institutional email addresses. Nurses and managers were given the option to complete the entire survey or specific sections of it if they preferred.

To preserve participant confidentiality, the collected data were anonymous and accessible solely to the principal investigators. Regular communication between local contact persons and the principal investigators was maintained to facilitate the exchange of participation updates.

### 2.5. Measurements

The survey collected socio-demographic information, which included age (in years), sex (male, female, or other), the highest level of education, overall work experience (in years), and work experience in the last service/ward (in years). Additionally, the survey aimed to assess the participants’ intentions to leave their current work setting, organization, or profession. This intention was measured using single-item measurement with binary response options: 1 (yes, I intend to leave the service within the next six months) and 2 (no, I do not intend to leave the service). Single-item measurements are a well-established technique for evaluating a concrete construct, such as the intention to leave [35].

In addition, to gather information on job satisfaction useful to test the criterion validity of the translated NMAs, participants were asked to rate their levels of satisfaction with their role, multidisciplinary work, leader, and organization using single items with a 5-point Likert scale. The scale ranged from 0 (very unsatisfied) to 4 (very satisfied). A higher score on the Likert scale indicates a higher level of satisfaction.

To assess nurse management actions that promote professional nurse autonomy, we used the translated NMAs derived from two previously published studies [13,30]. Permission to use was obtained from the authors. Of the translated version, two versions of the scale were created, one for nurse managers and another for nurses. Both versions consisted of 11 actions nurse managers could take to encourage autonomy. Participants were asked to rate the frequency with which nurse managers performed each action on a scale ranging from 1 (never) to 5 (always). The final score for the scale was calculated as the mean response across all items. Indeed, the final score of NMAs can be a number from 1 to 5, with higher scores indicating encouraging manager actions on developing nurse’s autonomy. In the original study, the scale had acceptable psychometric properties, an item-total correlation of 0.41–0.86, and an overall Cronbach’s alpha of 0.94, indicating high internal consistency [13,30].

### 2.6. Ethical Considerations

The study was conducted under ethical standards and principles outlined in the Helsinki Declaration [36]. The local Ethics Committee and Board of Directors at each participating center provided approval for the study. Before participation, all participants received information about the study and were required to sign an online informed consent form. Data access was limited exclusively to the research team to ensure participant confidentiality and data security.

### 2.7. Statistical Analysis

The study started by analyzing the characteristics of both nurses and nurse managers, using descriptive statistics and inferential comparisons to identify the distribution of respondents’ characteristics. Once the NMAs was translated and two versions were developed (one for nurses and one for nurse managers) [34], the validation process was conducted through MSA procedures separately in the nurses and nurse managers subgroups. The separate analytical approach in performing the MSA in nurses and nurse managers assumed that the final versions might have a different number of retained items. Through utilizing Loevinger’s H coefficient to gauge scalability and Molenaar–Sijtsma rho to appraise reliability, MSA incorporated robust statistical metrics that play a key role in the validation procedure. In this regard, Loevinger’s H coefficient serves as a critical indicator of scalability, helping to determine the hierarchical structure and coherence of the items within the scale and reflecting how well these items collectively represent the underlying construct. A higher H coefficient indicates that the items align well with the theoretical construct. On the other hand, Molenaar–Sijtsma rho is fundamental for evaluating the reliability of the measurement scale. This statistical measure quantifies the extent to which the items consistently and reliably capture the intended construct. A higher Molenaar–Sijtsma rho signifies stronger internal consistency, indicating that the items are reliably measuring the same underlying trait. While other indices (e.g., Cronbach’s alpha) assume that all items in a scale measure the same latent trait to the same degree and that the relationships among items are linear, Molenaar–Sijtsma rho is more flexible and specifically designed for use in non-parametric situations, such as the MSA.

The MSA was initially performed by employing the automatic item selection procedure to identify the most appropriate items for inclusion in the final Mokken scale separately for each subgroup. Then, the scalability coefficients for each item and the overall scalability of the scale were estimated in the two subgroups (H coefficient). Once the scalability was assessed, we checked whether each item in the datasets satisfies the monotonicity assumption, which is a requirement for Mokken scaling. Monotonicity means that as the level of the construct increases, the probability of endorsing a higher item response also increases. In other words, individuals who score high on the NMAs are expected to agree with all the items that individuals who score lower on the NMAs agree with, plus some additional items that are only endorsed by those with high trait levels. If an item violates the monotonicity assumption (maximum 80 violations can be accepted), the ordering of the item responses is inconsistent with the ordering of the underlying latent trait being measured. In this case, the item should be removed from the scale.

Following the monotonicity assessment, the invariant item ordering (IIO) procedure was used to determine whether the item hierarchy is consistent across different subgroups of the data. The IIO procedure tested whether the same items are ordered by the latent trait (i.e., the construct being measured) for different subgroups of respondents. If the ordering of items is invariant across subgroups, the Mokken scale is reliable and valid across different subgroups of respondents, such as different age groups, genders, or cultural backgrounds. On the other hand, if the item ordering is not invariant across subgroups, it may indicate that the scale is not measuring the same construct in different subgroups or that there are cultural or contextual differences in the interpretation of the items. However, the IIO procedure is not meant to assess multigroup measurement invariance. The procedure was conducted separately for data from nurses and nurse managers, and it involved several aspects. These aspects included calculating the scalability coefficient for each item in the scale, examining the acquiescence effect, detecting violations of the IIO procedure, and determining the maximum violation per item and the proportion of violations relative to the number of respondents. Additionally, the sum of scalability coefficients was computed, and the t-statistic associated with the maximum number of violations was compared against the critical value at alpha = 5%. In this stage, Loevinger’s H coefficient was calculated to determine the hierarchical order of the set of items in the scale, with values ranging from 0 to 0.3 indicating poor scalability, 0.3 to 0.4 indicating fair scalability, 0.4 to 0.5 indicating moderate scalability, 0.5 to 0.6 indicating good scalability, and values above 0.6 indicating excellent scalability. Finally, Molenaar–Sijtsma rho was used to assess the reliability or internal consistency of the scale.

When the scores of the NMAs were defined following the results of the MSA, in the context of criterion validity, the NMAs scores were assessed in relation to their ability to share linear relationships with external criteria. Following previous studies [7,10,14,21,23], it was hypothesized that NMAs shared positive correlations with satisfaction with the role, multidisciplinary work, leader, and organization and negative correlations with the intention to leave.

All analytical tests were conducted using R software, version 4.2.2 for Windows (R Core Team, 2022), with a significance level of 5% (alpha = 0.05). Missing data were less than 5% for each variable and managed with available case analysis. Hypothesis testing was performed using two-sided tests.

## 3. Results

### 3.1. Sample Characteristics

Table 1 describes the overall sample (n = 871) and the characteristics of the subgroup of nurses (n = 683) and nurse managers (n = 188). The majority of participants were from the northern regions of Italy (46.7%), with differences in the distribution between nurses and nurse managers (*p* < 0.001), and worked in public hospitals (71.2%) in both subgroups (*p* = 0.193). The mean age of the sample was 44.68 years (SD = 11.18), and the majority were female (75.1%). The mean work experience was 20.53 years (SD = 13.30), with nurse managers having significantly higher work tenure than nurses (*p* < 0.001). Regarding educational background, the majority of participants had a postgraduate certificate after a bachelor’s degree (45.7%), with higher rates among nurse managers (*p* < 0.001). A higher proportion of nurses intended to leave their ward/service (26.8%) and the organization/hospital (22.7%) compared to nurse managers (*p* < 0.01). The proportion of those with the intention to leave the profession was equal between the two groups (*p* = 0.080). There were also significant differences in satisfaction levels between the two groups, with nurse managers reporting higher satisfaction levels in multidisciplinary teamwork (*p* < 0.001), leadership (*p* < 0.001), and the organization/hospital (*p* < 0.001).

### 3.2. Mokken Scale Analysis

#### 3.2.1. Automatic Item Selection Procedure

The automatic item selection procedure (AISP) indicated in both samples (nurses and nurse managers) that all 11 items were adequate for being included in the Mokken scale. In nurses, the H coefficient was equal to 0.520 (standard error, SE = 0.017). In nurse managers, the H coefficient was 0.349 (SE = 0.032).

#### 3.2.2. Acquiescence and Monotonicity

In the subgroup of nurses, the items that were reported to be more susceptible to the acquiescence effect (i.e., a response bias that can occur in survey research where participants tend to answer regardless of their true beliefs or experiences) were item 6 (acquiescence index = 127), item 8 (acquiescence index = 123), and item 3 (acquiescence index = 106).

Overall, the acquiescence indexes ranged from 62 (item 2) to 127 (item 6). However, none of the items significantly violated monotonicity.

In nurse managers, the acquiescence effect was extremely limited. The acquiescence indexes ranged between 2 (items 1, 2, and 7) and 6 (item 5). None of the items significantly violated monotonicity.

#### 3.2.3. IIO Procedure and Reliability

The IIO procedure is shown in Table 2. In nurses, in the first step of the IIO procedure (*H^T^* = 0.420, Molenaar–Sijtsma rho = 0.90), items 7, 9, 10, 6, and 8 did not significantly contribute to the model’s explanatory power, violating the assumptions of the IIO. For this reason, in accordance with the authors that developed the scale, these items were removed in the second step, and the scale showed improved scalability (*H^T^* = 0.630, Molenaar–Sijtsma rho = 0.890).

In the nurse managers subgroup, a single step of IIO was satisfactory (*H^T^* = 0.620, Molenaar–Sijtsma rho = 0.830); no significant violations of the IIO were detected, and all the items were retained for the scoring procedure.

At the end of the IIO procedure, items 1, 2, 3, 4, 5, and 11 were used to compute the score of the NMAs for nurses, while all 11 items were used for scoring the NMAs for nurse managers.

**Figure 1:**
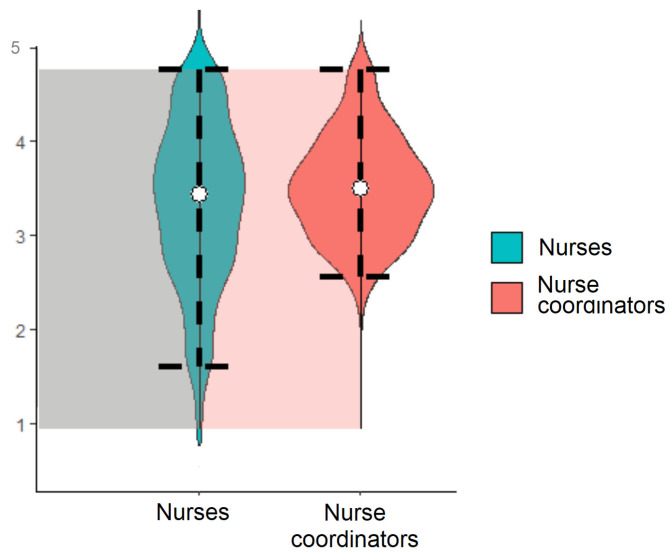
Violin plots describing the distribution of the NMAs scores in nurses (items 1, 2, 3, 4, and 5) and nurse managers (all the items).

Figure 1 shows the distribution of the two scores in nurses (median = 3.67; interquartile range, IQR = 3–4.17) and nurse managers (median = 3.73; IQR = 3.36–4.09).

### 3.3. Criterion Validity

In the lower part of the table, under the diagonal, Table 3 shows the correlations between the NMAs scores obtained in the subsample of nurses (computing only the retained items) with the satisfaction scores and intention to leave. The a priori hypotheses of positive linear relationships with satisfaction scores and negative relationships with the intention to leave were met. In the upper part of the table, above the diagonal, Table 3 shows the correlations between the NMAs scores obtained in the subsample of nurse managers (computing all items) with the satisfaction scores and intention to leave. The a priori hypotheses were partially met by considering the significant correlation between NMAs score and satisfaction regarding the current role (r = 0.279; *p* = 0.048) and the higher NMAs reported by nurse managers without intention to leave the ward (r_pb_ = 0.236; *p* = 0.053).

## 4. Discussion

The present study aimed to evaluate items’ scalability of NMAs as well as its validity and reliability among nurses and nurse managers in the Italian healthcare context. This study was necessary to ensure that the instrument is suitable for use in this specific cultural context. The findings of the study showed that the NMAs was a valid and reliable instrument to measure nurse manager actions in the Italian healthcare context.

More precisely, the MSA confirmed that the Italian NMAs has a version for nurses encompassing six items (items from 1 to 6 and item 11 of the original scale) and a version for nurse managers that retained all 11 items as per the original scale [13,30]. Before the current study, only two prior research studies partially explored the psychometric properties of the NMAs within nursing samples [13,30]. However, none of these studies have specifically examined the NMAs in a sample of nurse managers. In these studies, the scale was found to have an item-total correlation of 0.41–0.86 [30] and 0.48–0.86 [13] and an overall Cronbach’s alpha of 0.94, indicating high internal consistency [13,30]. No other construct validity testing has been performed. The current study, through the utilization of the MSA, has contributed substantially to the enhancement of the NMAs’ internal validity, particularly in comparison to prior research that predominantly relied on Cronbach’s alpha and item-total correlations [13,30]. While divergent underlying assumptions and methodologies preclude direct comparisons between Molenaar–Sijtsma rho and Cronbach’s alpha or item-total correlation employed in previous studies, our study’s methodology introduces an innovative paradigm for establishing the scale’s reliability in relation to its internal consistency. Notably, the two distinct versions tailored for nurses and nurse managers demonstrated robust internal validity that paves the way for future applications.

In fact, it is essential to explore both nurse managers’ self-evaluations and nurses’ perceptions regarding these actions to achieve a comprehensive understanding of how nurse managers’ actions aimed at promoting nurses’ autonomy work in practice. With this rationale, two versions of the scale were created in Italian, maintaining the original scale’s intended meaning while differing only in the phrasing at the beginning of each item; “my nurse manager” for the nurse version and “I” for the nurse manager version.

Nurse managers and nurses hold distinct roles within a healthcare organization, each contributing unique insights and experiences. Collecting data from both sources offers several benefits. This dual approach enables researchers to attain a more holistic perspective on various aspects of leadership and patient care; to identify potential communication and collaboration gaps or barriers, to define areas for improvement that contribute to a more healthful and productive workplace, to inform strategies aimed at improving leadership effectiveness and increasing employee satisfaction. Ultimately, the ability to gather data from both nurse managers and nurses allows for a more versatile utilization of the research results in both academic studies and practical clinical applications.

During the validation process, it was established that the two Italian versions of the NMAs contained a different number of items. The items that were removed from the nurse version pertained to the promotion of professional autonomy in specific areas, such as granting the ability to self-schedule shifts (item 6), stimulating intellectual discussions about work (item 7), delegating 24 h responsibility about unit decisions (item 8), helping the group develop plans to meet their educational needs (item 9), and encouraging participation in research projects and use of research (item 10). All of these items are related to nurse autonomy in making decisions concerning the unit. Previous studies on the topic have documented that nurse managers prioritized fostering nurses’ autonomy concerning patient care choices rather than operational decisions within the unit [13,18,30]. This managerial mindset can be adopted by nurses, resulting in perceptions that nurses’ decision-making importance in these areas is diminished. Additionally, the violation of IIO for those items in the final version developed for nurses might arise from potential differences in how they perceive situations compared to nurse managers. Considering the viewpoint of nurses, it is plausible that the above-mentioned aspects of professional autonomy could be influenced by the national context of the nursing profession, characterized by limited decision-making authority and autonomy [24,27], as well as factors like nursing shortages [1], increased workloads [4], nurse educational background and tenure [30], nurse manager leadership [12,18], nature of work environments [30], or organizational dynamics [7,12]. Furthermore, the presence of shortcomings in the scale cannot be excluded [30]. Therefore, further research is needed to delve into these variations in perception between nurses and nurse managers and to ascertain how these differences might impact the validity of the NMAs.

This study employed hypothesis testing to examine the construct validity of the NMAs scores in relation to external criteria: satisfaction levels and intention to leave. The results confirmed the validity of NMAs scores in the Italian context, as they exhibited positive correlations with satisfaction with the role, multidisciplinary teamwork, leader, and organization and negative correlations with the intention to leave, as hypothesized based on previous studies [7,10,14,21,23]. While these hypotheses were fully accepted in the subsample of nurses, they were only partially accepted in relation to the nurse managers. Indeed, the NMAs scores exhibited a positive correlation with nurse managers’ satisfaction with their role and a negative correlation with their intention to leave the unit/ward. These differences might reflect the limited sample size of nurse managers or may be linked to the favorable outcomes associated with effective nurse manager leadership and well-being in the work context. In the existing literature, the exploration of nurse managers’ satisfaction and intention to leave has received comparatively less attention than in the literature regarding nursing staff. A recent systematic review [37] underscored autonomy, driving positive change, social support, team cohesion, and well-being as important factors for nurse manager job satisfaction. Similarly, strong inter-organizational relationships and support, professional growth, and top management endorsement have been recognized as influential aspects of retention [38,39]. Given the pivotal role that nurse managers play in ensuring nurses’ retention and well-being, along with their substantial impact on the success of healthcare organizations [37], it becomes crucial to conduct an in-depth exploration of the phenomenon related to nurse managers as well.

The performed validation study has implications for nursing practice and research in Italy and internationally. The use of the NMAs in healthcare settings can help identify the actions and behaviors of nurse managers that enhance professional autonomy among nurses. This information can be used to identify areas for managerial improvement, such as stimulating nurses’ autonomous decision-making on patient care, enhancing multidisciplinary teamwork, promoting nurses’ participation in unit operational decisions, and ultimately evaluating its effects on the quality of patient care [13,30]. In addition, the NMAs could be used to evaluate the effectiveness of these interventions and identify further areas that could benefit from educational programs or other interventions. Using the NMAs also has possible indirect implications for clinical practice, as it enables the determination of associations with job satisfaction, intention to leave, enhanced multidisciplinary work, and the effectiveness of leadership style [18,40].

Considering the intricate nature of the current healthcare environment [28], fostering and upholding nurse autonomy becomes a noteworthy challenge. More dialogues should be initiated among nurses and nurse managers to devise strategies to enhance nurses’ autonomy through environmental modifications. Furthermore, the NMAs scale provides a valuable avenue for investigating impediments that impede autonomous decision-making among nurses while also facilitating the cultivation of their engagement in unit-level operational choices. This initiative strives to foster a collaborative environment between nurses and physicians, underpinned by mutual trust, respect, and the synergistic integration of expertise, competencies, and ethical values [13,18,41].

At an international level, the results of this study can contribute to the development of standardized measures for nurses and nurse managers that could be used across different countries and settings. In fact, the use of hypothesis testing and MSA in this study provides a model for other researchers to follow when assessing the validity and reliability of similar measures in different populations. It is likely that the obtained results might be consistent in future studies performed in other cultural settings. Overall, this study can inform nursing practice and research in other countries and settings and contribute to the development of more rigorous and culturally relevant assessment tools in the field of nursing.

The current study is not without limitations. Firstly, the cross-sectional design employed for data collection prevents causal relationships from being established in the hypothesis testing for construct validity. Secondly, the sample size for nurse managers was relatively small, potentially limiting the findings’ generalization and the ability to detect significant correlations in the hypothesis testing. Additionally, the study was conducted only in Italy, which may restrict the generalizability of the findings to other settings. Despite these limitations, the study has notable strengths. The adaptation and translation process of the NMAs for the Italian context was rigorously conducted, and the psychometric validation was accurate, bolstering the instrument’s validity for use in Italy. Furthermore, the study employed a hypothesis testing approach to test the criterion validity of the NMAs scores, enhancing the conclusions that can be drawn from the findings.

Future research should focus on psychometric testing using larger samples of nurse managers and nurses to re-test the shortened version, establish dimensionality using factor analysis, assess the measurement invariance in both subgroups and integrate the test-retest analysis to affirm the measurement reliability. In upcoming investigations, adopting longitudinal research designs is strongly advised to enhance the understanding of the NMAs scale’s stability and potential fluctuations over time. Such designs allow for tracking the consistency of responses and patterns of NMAs scores across different time points, offering insights into the temporal reliability and validity of the scale. By capturing potential variations over time, researchers can better understand the NMAs’ stability, allowing for more accurate interpretations of its effects on nurses’ autonomy and work environments. Additionally, a promising avenue for future research involves examining the cross-cultural invariance of the NMAs across diverse populations of nurses and managers from various countries. This approach could help uncover potential cultural nuances that might influence how nurse managers’ actions are perceived and impact nurses’ autonomy. Assessing the scale’s cross-cultural validity could help researchers ensure that the NMAs’ constructs hold true across different cultural contexts, thus enhancing the scale’s generalizability and applicability globally. Furthermore, empirical investigations that delve into the dyadic or multilevel dynamics between nurse managers and nurses and how these interactions influence both nurse and patient outcomes hold significant promise. A comprehensive understanding of the interplay between nurse manager actions, nurses’ autonomy, and overall healthcare outcomes could be gained by employing the two versions of the scale. This approach may shed light on the intricate relationships that influence nurse and patient experiences, potentially guiding interventions aimed at enhancing the quality of care and work environments.

## 5. Conclusions

NMAs is a valid and reliable instrument for measuring the actions of nurse managers in the Italian healthcare system and can be used to identify areas for improvement in professional autonomy among nurses and nurse managers. This study evaluated the validity and reliability of the NMAs in the Italian context and conducted a psychometric validation among nurses and nurse managers, developing two different versions. The results from this study demonstrate the establishment of unidimensional hierarchies, robust scalability coefficients, and noteworthy internal consistency for both the nurses’ and nurse managers’ groups. This MSA-based evaluation reinforces the reliability and validity of the scale, affirming its potential utility in work contexts and research endeavors. Other implications include recognizing the potential advantages of employing NMAs as a guide to foster nurses’ autonomy and provide decision-making support by serving as an evaluation tool to identify managerial and nurse autonomy weak points for targeted intervention implementation. NMAs may also aid in assessing leadership’s effectiveness in reinforcing multidisciplinary teamwork, promoting nurse well-being and satisfaction, and influencing intentions to leave the unit or profession. While this study provides valuable insights into the importance of professional autonomy for nurses and nurse managers in the Italian context and how the employed approach and results can inform future research and practice, further empirical studies are required.

## Figures and Tables

**Table 1:** Sample characteristics (nurses and nurse managers).

		Overall (N =871)		Nurses (N = 683)		Nurse Managers (N = 188)		*p*
		N	%	N	%	N	%	
Region								
	Northern regions	407	46.7	337	49.3	70	37.8	<0.001
	Central regions	161	18.5	134	19.3	27	14.4	
	Southern regions	303	34.8.	212	31	91	47.9	
Setting								
	Public hospital	620	71.2	479	70.1	141	75	0.193
	Primary care service	117	13.4	91	13.3	26	13.8	
	Private hospital delivering public service	134	15.4.	113	16.5	21	11.2	
Age								
	Years (mean; SD)	44.68	11.18	42.57	11.25	52.35	6.65	<0.001
Sex								
	Female	654	75.1	510	75	144	76.6	0.676
Work experience								
	Years (mean; SD)	20.53	13.30	16.94	11.14	30.42	8.69	<0.001
Work experience in the last ward/service								
	Years (mean; SD)	8.44	8.50	8.32	8.3	8.85	9.12	0.277
Educational background								
	BSc or equivalent title	191	21.9	181	26.5	10	5.4	<0.001
	Postgraduate certificate after BSc	398	45.7	286	41.9	112	59.6	
	Master of Science	199	22.8	162	23.7	37	19.7	
	Postgraduate certificate after MSc	72	8.3	44	6.4	28	14.9	
	Other postgraduate education	1	0.1	1	0.1	0	0	
	PhD	10	1.1	9	1.3	1	0.5	
Intention to leave the ward/service								
	Yes	214	24.6	183	26.8	31	16.5	0.004
Intention to leave the company/hospital								
	Yes	184	21.1	155	22.7	29	15.4	0.031
Intention to leave the nursing profession								
	Yes	143	16.4	120	17.6	23	12.2	0.080
Satisfaction regarding the current role								
	Score (0 = completely not satisfied; 4 = completely satisfied) (median; IQR)	2	3–3	3	2–3	3	2–4	0.138
Satisfaction regarding multidisciplinary work								
	Score (0 = completely not satisfied; 4 = completely satisfied) (median; IQR)	2	3–3	3	2–3	3	3–3	0.001
Satisfaction regarding the leadership								
	Score (0 = completely not satisfied; 4 = completely satisfied) (median; IQR)	2	3–4	3	2–4	3	3–3	<0.001
Satisfaction with the company/hospital								
	Score (0 = completely not satisfied; 4 = completely satisfied) (median; IQR)	2	3–3	3	2–3	3	2–3	<0.001

Legend: SD = standard deviation; BSc = Bachelor of Sciences (in Nursing); MSc = Master of Sciences (in Nursing); IQR = interquartile range.

**Table 2:** Invariant item ordering procedure in the Mokken Scale Analysis.

**Nurses (N = 683)**															
**Step**	**Items**	**Mean (SD)**	**H**	**#ac**	**#vi**	**#vi/#ac**	**maxvi**	**sum**	**sum/#ac**	**tmax**	**#tsig**	**crit**	**Selection**	***H^T^***	**Rho**
Step 1	NMAs2	3.98 (1.02)	0.57	70	1	0.01	0.13	0.13	0.0019	0.83	0	11	0	0.42	0.90
	NMAs1	3.97 (1.04)	0.56	69	1	0.01	0.13	0.13	0.0019	0.83	0	12	0		
	NMAs5	3.74 (1.08)	0.58	70	2	0.03	0.13	0.27	0.0038	0.98	0	17	0		
	NMAs4	3.71 (1.05)	0.58	70	1	0.01	0.13	0.13	0.0019	0.93	0	12	0		
	NMAs3	3.52 (1.22)	0.53	70	2	0.03	0.17	0.32	0.0046	1.26	0	26	0		
	NMAs7	3.48 (1.14)	0.61	71	4	0.06	0.34	0.88	0.0124	2.63	2	77	2		
	NMAs9	3.46 (1.14)	0.60	68	4	0.06	0.23	0.61	0.0090	1.76	1	52	1		
	NMAs10	3.37 (1.22)	0.54	70	2	0.03	0.37	0.56	0.0081	2.94	1	70	1		
	NMAs6	2.95 (1.10)	0.39	68	6	0.09	0.37	1.36	0.0200	2.94	3	111	3		
	NMAs8	2.72 (1.16)	0.31	69	4	0.06	0.26	0.82	0.0119	2.01	1	76	1		
	NMAs11	2.45 (1.31)	0.45	71	1	0.01	0.23	0.23	0.0032	1.32	0	31	0		
Step 2	NMAs2	3.98 (1.02)	0.65	33	1	0.03	0.13	0.13	0.0038	1.14	0	12	0	0.63	0.89
	NMAs1	3.97 (1.04)	0.64	32	1	0.03	0.13	0.13	0.0040	1.14	0	12	0		
	NMAs5	3.74 (1.08)	0.66	33	1	0.03	0.18	0.18	0.0055	1.44	0	21	0		
	NMAs4	3.71 (1.05)	0.66	30	1	0.03	0.18	0.18	0.0061	1.44	0	22	0		
	NMAs3	3.52 (1.22)	0.61	33	0	0.00	0.00	0.00	0.0000	0.00	0	0	0		
	NMAs11	2.45 (1.31)	0.40	25	0	0.00	0.00	0.00	0.0000	0.00	0	0	0		
**Nurse managers (N = 188)**															
**Step**	**Items**	**Mean (SD)**	**H**	**#ac**	**#vi**	**#vi/#ac**	**maxvi**	**sum**	**sum/#ac**	**tmax**	**#tsig**	**crit**	**Selection**	***H^T^***	**Rho**
Step 1	NMAs2	4.42 (0.54)	0.31	10	0	0	0	0	0	0	0	0	0	0.62	0.83
	NMAs1	4.62 (0.56)	0.32	10	0	0	0	0	0	0	0	0	0		
	NMAs5	4.20 (0.72)	0.40	10	0	0	0	0	0	0	0	0	0		
	NMAs4	4.09 (0.77)	0.36	10	0	0	0	0	0	0	0	0	0		
	NMAs3	4.01 (0.93)	0.36	11	0	0	0	0	0	0	0	0	0		
	NMAs7	3.96 (0.81)	0.42	10	0	0	0	0	0	0	0	0	0		
	NMAs9	3.82 (0.89)	0.44	10	0	0	0	0	0	0	0	0	0		
	NMAs10	3.78 (0.98)	0.37	10	0	0	0	0	0	0	0	0	0		
	NMAs6	2.65 (1.27)	0.31	10	0	0	0	0	0	0	0	0	0		
	NMAs8	2.75 (1.19)	0.29	11	0	0	0	0	0	0	0	0	0		
	NMAs11	2.75 (1.24)	0.30	10	0	0	0	0	0	0	0	0	0		

Legend: H: This column contains the scalability coefficient for each item in the scale. The scalability coefficient represents the extent to which items in the scale form a unidimensional hierarchy, with higher values indicating a stronger item hierarchy. #ac: This column represents the number of respondents who equally answered each item in the scale. This is sometimes referred to as the “acquiescence” column. #vi: This column represents the number of violations of the IIO property for each item. An item violates the invariant item ordering property if it has a lower scalability coefficient than a higher-ranked item. #vi/#ac: This column represents the proportion of violations relative to the number of respondents who answered the item. maxvi: This column represents the maximum number of violations for any item in the scale. sum: This column represents the sum of the scalability coefficients for each item in the scale. sum/#ac: This column represents the average scalability coefficient across all items in the scale, normalized by the number of respondents who answered each item. tmax: This column represents the t-statistic associated with the maximum number of violations. #tsig: This column represents the number of items for which the t-statistic exceeds the critical value, indicating a significant violation of the invariant item ordering property. crit: This column represents the critical value of the t-statistic at the specified level of significance equal to 0.05. If the t-statistic exceeds this value, the violation of the invariant item ordering property is considered statistically significant. Therefore, zero means t-statistic > 0.05 (non-significant violations). Selection = Backward selection: this is a method used in statistical modeling to eliminate predictors from a model that do not significantly contribute to the model’s explanatory power. *H^T^* = Loevinger’s *H* coefficient, which measures the extent to which a set of items in a scale form a hierarchical order. Rho = Molenaar–Sijtsma statistic of reliability. SD = standard deviation.

**Table 3:** Correlations for the hypotheses testing.

	(1)	(2)	(3)	(4)	(5)	(6)	(7)	(8)
Score NMAs (1)	–	0.279 *	−0.014	0.071	0.022	0.236 *	−0.032	0.071
Satisfaction regarding the current role (2)	0.277 **	–	0.652 **	0.580 **	0.586 **	0.345 **	0.247 **	0.266 **
Satisfaction regarding multidisciplinary work (3)	0.369 **	0.604 **	–	0.623 **	0.526 **	0.170 *	0.048	0.137
Satisfaction regarding the leadership (4)	0.533 **	0.459 **	0.575 **	–	0.632 **	0.138	0.251 **	0.195 **
Satisfaction with the company/hospital (5)	0.302 **	0.560 **	0.481 **	0.495 **	–	0.275 **	0.478 **	0.286 **
Intention to leave the ward/service (6)	0.183 **	0.359 **	0.300 **	0.261 **	0.287 **	–	0.406 **	0.14
Intention to leave the company/hospital (7)	0.210 **	0.328 **	0.297 **	0.313 **	0.483 **	0.390 **	–	0.290 **
Intention to leave the nursing profession (8)	0.158 **	0.325 **	0.248 **	0.150 **	0.366 **	0.285 **	0.365 **	–

Note: The upper side of the table shows the correlations derived from a subsample of nurse managers (N = 188) by using a nurse managers’ action (NMAs) score computed with all 11 items of the scale. The lower side of the table shows the correlations derived from a subsample of nurses (N = 683) by using a score computed with items 1, 2, 3, 4, 5, and 11 of the NMAs scale. The positive point-biserial correlations of intention to leave (variables 6, 7, and 8) have to be interpreted as negative linear relationships because the intention to leave was coded as 1 = yes and 2 = no, indicating that higher scores of NMAs and satisfaction were associated with “no intention to leave”. ** indicates *p* < 0.01; * indicates *p* < 0.05

## Data Availability

The data presented in this study are available on reasonable request from the corresponding author.

## Supplementary Materials

The following supporting information can be downloaded at: https://www.mdpi.com/article/10.3390/nursrep13030102/s1, Table S1: GRRAS checklist for reporting of studies of reliability and agreement.

## Public Involvement Statement

No public involvement in any aspect of this research.

## Guidelines and Standards Statement

This manuscript was drafted against the Guidance for Reporting Involvement of Patients and the Public-GRRAS checklist [42] available in supplementary file, Table S1.

## Appendix A

In the appendix, you can find the original English and the Italian versions of the scale Nurse Manager Actions. Table A1 (Italian scale—nurse version) and Table A2 (Italian scale—nurse manager version).

**Table A1:** NMAs—Italian version for nurses.

Quanto frequentemente il suo coordinatore usa le seguenti azioni per promuovere l’autonomia del proprio gruppo di infermieri. Indichi quale risposta rappresenta al meglio la sua opinione.						
	Il mio coordinatore infermieristico…	Non so	Raramente	A volte	Spesso	Sempre
1	Incoraggia il gruppo a comunicare apertamente con tutti i membri del team sanitario. *(Encourage nurses to communicate openly with all members of the healthcare team)*	1	2	3	4	5
2	Supporta il gruppo nella risoluzione dei conflitti con medici, pazienti e colleghi. *(Support nurses to resolve conflicts with physicians, patients, and colleagues)*	1	2	3	4	5
3	Incoraggia la leadership tra gli infermieri. (Encourage leadership among nurses)	1	2	3	4	5
4	Supporta il processo decisionale autonomo del gruppo. *(Support nurses’ autonomous decision-making)*	1	2	3	4	5
5	Consulta il gruppo mentre stabilisce gli standard di cura. *(Consult nurses while establishing standards of care)*	1	2	3	4	5
11	Coinvolge il gruppo nella pianificazione delle spese capitali. *(Involve nurses in planning the capital expenditures)*	1	2	3	4	5

**Table A2:** NMAs—Italian version for nurse managers.

Quanto frequentemente usa le seguenti azioni per promuovere l’autonomia del suo gruppo. Indichi la risposta che meglio rappresenta la sua opinione.						
	Promozione dell’autonomia del gruppo	Mai	Raramente	A volte	Spesso	Sempre
1	Incoraggio il gruppo a comunicare apertamente con tutti i membri del team sanitario. *(Encourage nurses to communicate openly with all members of the healthcare team)*	1	2	3	4	5
2	Supporto il gruppo nella risoluzione dei conflitti con medici, pazienti e colleghi. *(Support nurses to resolve conflicts with physicians, patients, and colleagues)*	1	2	3	4	5
3	Incoraggio la leadership tra gli infermieri. *(Encourage leadership among nurses)*	1	2	3	4	5
4	Supporto il processo decisionale autonomo del gruppo. *(Support nurses’ autonomous decision-making)*	1	2	3	4	5
5	Consulto il gruppo mentre stabilisco gli standard di cura. *(Consult nurses while establishing standards of care)*	1	2	3	4	5
6	Consento agli infermieri di auto-programmare i turni. *(Allow nurses to self-schedule)*	1	2	3	4	5
7	Stimolo le discussioni intellettuali del gruppo sul lavoro. *(Stimulate nurses’ intellectual discussions about work)*	1	2	3	4	5
8	Delego al gruppo la responsabilità su 24 ore delle decisioni sul reparto. *(Delegate to nurses 24-h responsibility about their units’ decisions)*	1	2	3	4	5
9	Aiuto il gruppo a sviluppare piani per soddisfare i loro bisogni educativi. *(Help nurses to develop plans to meet their educational needs)*	1	2	3	4	5
10	Incoraggio il gruppo a partecipare a progetti di ricerca e utilizzare la ricerca. *(Encourage nurses to participate in research projects and use research)*	1	2	3	4	5
11	Coinvolgo il gruppo nella pianificazione delle spese capitali. *(Involve nurses in planning the capital expenditures)*	1	2	3	4	5
