# Supplementary material for: Assessment of the Psychometric Characteristics of the Italian Version of the Nurse Manager Actions Scale
Source: Nurs Rep. 2023 Sep 1;13(3):1185–202. doi: 10.3390/nursrep13030102 (PMC10534939; doi:10.3390/nursrep13030102)
Supplement: Supplementary file 1 [file nursrep-13-00102-s001.zip › nursrep-2509473-supplementary.pdf]

Supplementary material

Table A3. GRRAS checklist for reporting of studies of reliability and agreement

Version based on Table I in: Kottner J, Audigé L, Brorson S, Donner A, Gajeweski BJ, Hróbjartsson A, Robersts C, Shoukri M, Streiner DL. Guidelines for reporting reliability and agreement studies (GRRAS) were proposed. *J Clin Epidemiol*. **2011**, 64(1):96-106

**Section**	**Item** #	**Checklist item**	**Reported on page** #
Title/Abstract	1	Identify in title or abstract that interrater/intrarater reliability or agreement was investigated.	The study was identified as a validation study in the title
Introduction	2	Name and describe the diagnostic or measurement device of interest explicitly.	2-3
	3	Specify the subject population of interest.	2-3
	4	Specify the rater population of interest (if applicable).	2-3
	5	Describe what is already known about reliability and agreement and provide a rationale for the study (if applicable).	3-4
Methods	6	Explain how the sample size was chosen. State the determined number of raters, subjects/objects, and replicate observations.	4-6
	7	Describe the sampling method.	4-6
	8	Describe the measurement/rating process (e.g. time interval between repeated measurements, availability of clinical information, blinding).	4-6
	9	State whether measurements/ratings were conducted independently.	4-6
	10	Describe the statistical analysis.	6-7
Results	11	State the actual number of raters and subjects/objects which were included and the number of replicate observations which were conducted.	7-13
	12	Describe the sample characteristics of raters and subjects (e.g. training, experience).	7-13
	13	Report estimates of reliability and agreement including measures of statistical uncertainty.	7-13
Discussion	14	Discuss the practical relevance of results.	13-16
Auxiliary material	15	Provide detailed results if possible (e.g. online).	Grras checklist supplementary
